# The Impact of Failures and Successes on Affect and Self-Esteem in Young and Older Adults

**DOI:** 10.3389/fpsyg.2019.01795

**Published:** 2019-08-06

**Authors:** Alessia Rosi, Elena Cavallini, Nadia Gamboz, Tomaso Vecchi, Floris Tijmen Van Vugt, Riccardo Russo

**Affiliations:** ^1^Department of Brain and Behavioral Sciences, University of Pavia, Pavia, Italy; ^2^Laboratory of Experimental Psychology, Suor Orsola Benincasa University, Naples, Italy; ^3^IRCCS Mondino Foundation, Pavia, Italy; ^4^Department of Psychology, McGill University, Montreal, QC, Canada; ^5^Haskins Laboratories, New Haven, CT, United States; ^6^Department of Psychology, University of Essex, Colchester, United Kingdom

**Keywords:** aging, individual differences, affect, self-esteem, success-failure manipulation

## Abstract

Older adults are assumed to change their affect states in reaction to positive and negative stimuli across the life span. However, little is known about the impact of success and failure events on age-related changes in affect states and, particularly, in self-esteem levels. To fill this gap in the literature, in the present study changes in affect and self-esteem in 100 young (19–30 years) and 102 older adults (65–81 years) were assessed after participants experienced success and failure in a demanding cognitive task. Overall, the success-failure manipulation induced changes on affect states and on state self-esteem, not on trait self-esteem. Regarding age differences, older and young adults were affected to the same extent by experiences of successes and failures. Theoretical considerations of the empirical findings are provided in the general discussion.

## Introduction

Successes and failures may influence mood, affect and emotional states to varying degrees (Nummenmaa and Niemi, 2004), as well as self-esteem levels insofar these events are perceived as personal successes or failures (Crocker and Wolfe, 2001). Affect and self-esteem are linked in everyday experience, such that people with high self-esteem report more positive affect and people who lack self-worth are generally in negative mood (e.g., Lyubomirsky et al., 2006). Given that normal aging is associated with changes in emotional experience (Carstensen et al., 1999) and in self-esteem levels (Orth and Robins, 2014), it is of interest to assess whether experimentally induced successes and failures differently impact on affect and self-esteem of young and older adults.

Affect is a broad psychological construct that refers to mood and emotional states associated to what people feel in reaction to what is happening (Gray and Watson, 2007). Affect can be structured along positive and negative dimensions. Positive affect represents the level of pleasant engagement comprising positively valenced emotional states (Watson and Tellegen, 1985). Negative affect reflects a feeling of unpleasant engagement thus revealing an individual’s reaction to experiencing some type of negative emotional states (Watson and Tellegen, 1985).

Several cross-sectional and longitudinal studies have shown that affective reactions to positive and negative stimuli change across the life-span (e.g., Carstensen et al., 2006; Kliegel et al., 2007; Röcke et al., 2009). The socioemotional selectivity theory (SST; Carstensen et al., 1999) explains this change by arguing that, with increasing age, life expectancy is perceived as more limited and, consequently, the reduced time perspective leads older adults to shift their motivational goals to optimize positive affective experiences (Carstensen et al., 2006). In line with this perspective, other researchers explain age-related changes in affective experience in terms of affect-regulatory responses (Gross et al., 1997; Labouvie-Vief and Medler, 2002; Blanchard-Fields et al., 2004). According to these theoretical frameworks, older adults are better equipped to regulate their affect states and use this regulatory ability to manage positive and negative emotional states in order to avoid negative affect responses and to optimize the positive ones (Gross et al., 1997; Labouvie-Vief and Medler, 2002). An alternative view imputes lifespan changes in affective experiences to an age-related decline in physiological reactivity (Levenson et al., 1991). This decline should lead older adults to respond to emotional stimuli with lower emotional impact than younger adults. For example, it has been reported that older adults show a reduced physiological arousal when watching emotional films than younger adults (Tsai et al., 2000) and when discussing about conflictual issues with their spouses (Levenson et al., 1994). Hence, according to the reduced physiological reactivity perspective, older adults would be expected to be less physically and affectively/emotionally reactive than younger adults to both positive and negative events.

Self-esteem is a self-evaluation construct corresponding to an overall view of what people contemplate and evaluate about themselves (Baumeister, 1998) and can be characterized either as a trait or as a state (Heatherton and Polivy, 1991). Trait self-esteem represents a stable evaluation of the self, while state self-esteem is a context-specific state of self-worth that can fluctuate in reaction to situational factors (Crocker and Wolfe, 2001).

Cross-sectional and longitudinal studies have reported that self-esteem tends to show age-related changes from young to old age (e.g., Robins et al., 2002; Orth et al., 2012). This body of research has focused on developmental changes in the level of trait self-esteem, and only very few studies investigated age-related changes in state self-esteem (Meier et al., 2011).

Regarding trait self-esteem, a number of longitudinal studies found that it tends to increase from adolescence to midlife, with a peak at about 50–60 years, and then decreases in old age (e.g., Coleman et al., 1993; Orth et al., 2010, 2012; Wagner et al., 2013). This age-related decrease could derive from the experiences of losses in domains on which people may have staked their self-esteem, such as a loss of social roles, work, relationships, health, and attractiveness (Crocker and Wolfe, 2001; Robins et al., 2002; Orth et al., 2010). Other studies, however, found that trait self-esteem remains relatively stable in old age (e.g., Collins and Smyer, 2005; Huang, 2010; Wagner et al., 2013), suggesting that older adults adopt self-regulation strategies and have adaptive capacities to protect their self-worth from experiences of loss (Charles and Carstensen, 2010; Wagner et al., 2013).

Regarding the state self-esteem, Crocker and Wolfe (2001) hypothesized that, with increasing age, self-esteem becomes more stable and less susceptible to the impact of external daily life events. A recent study supported this hypothesis: Meier et al. (2011) found that from 13 to 72 years of age, short-term fluctuations in state self-esteem in daily life become better adjusted, i.e., more stable and less susceptible to self-relevant events with increasing age. Furthermore, state self-esteem in older adults appears to be affected by stressful life events (Liu et al., 2014) and financial losses (Krause et al., 1991), while, in young adults, it appears to be affected by academic and sport-related successes and/or failures (Hines and Groves, 1989; Crocker and Luhtanen, 2003).

An extensive body of research has examined how the trajectory of affect and self-esteem undergoes changes across the lifespan. However, only few studies aimed at directly assessing the extent to which positive and negative events may differentially impact on affect and self-esteem in young and older adults. For instance, using a longitudinal daily experience design, Röcke et al. (2009) showed that older adults displayed significantly lower levels of affect variability than young adults in association to both positive and negative events over a period of 45 days. Currier (1993), in an 18-month long longitudinal study design, found that positive events increased self-esteem, while negative events reduced self-esteem among older adults. These methodologies, although ecological, do not allow researchers to assess the causal impact of positive and negative events on affect and self-esteem. Regarding affect manipulation, Mood Induction Procedures (MIPs; see Gerrards-Hesse et al., 1994, for a review) allows to experimentally induce changes in affect states by inducing the experience of a particular emotion. Success-Failure Manipulations (SFMs; for a meta-analysis see Nummenmaa and Niemi, 2004) is another kind of manipulation that induces changes not only in affect states but also in self-esteem through the experience of a success or a failure event. These two methodologies are quite different because in the latter one (SFM) mood modifications may occur also because of self-esteem changes. Results on age-related changes in affect state obtained using MIPs are quite controversial as either equivalent affect states changes in young and older adults (e.g., Knight et al., 2002; Phillips et al., 2002), or an increased emotional variability in older adults than in young adults (Kunzmann and Grühn, 2005; Kliegel et al., 2007) have been reported. Studies using the SFM with young adults usually reported a tendency to show an increase in positive affect and self-esteem and a decrease in negative affect following a success (e.g., Krohne et al., 2002; Henkel and Hinsz, 2004; Franken et al., 2006; Bongers et al., 2009; Sekścińska, 2015). However, to the best of our knowledge, only one study used the SFM to directly compare changes in affect and self-esteem states in young and older samples (Rosi et al., 2016) using a manipulated gambling task inducing success and failure experiences. Results showed that emotional responses displayed by older adults were less extreme than those displayed by young adults. No age differences were obtained regarding the negative affect nor in self-esteem. In this last measure, the lack of significant difference probably occurred because the questionnaire used by Rosi et al. (2016) to assess trait self-esteem was not adequate and sensitive enough to assess changes due to the experimental manipulation. The pattern of results obtained by Rosi et al. (2016) appears consistent with the reduced physiological reactivity perspective that predicts older adults to respond to emotional stimuli with lower emotional impact than younger adults.

The aim of the present study is to investigate the experience of success or failure in a more demanding cognitive task and by using more specific measures of affect states and self-esteem than Rosi et al. (2016). Specifically, in the present study, participants had to solve a selection of the Raven’s Advanced Progressive Matrices (APM; Raven et al., 1988) manipulated in terms of items’ difficulty. Young and older adults randomly assigned to the success condition performed a selection of easy items and received a positive feedback on their performance. Conversely, participants assigned to the failure condition performed a selection of difficult items and received a negative feedback on their performance. As older adults have more stereotypes and negative beliefs about their cognitive performance than young adults (e.g., Lineweaver and Hertzog, 1998; Chasteen et al., 2005), it seems feasible to assume that the success-failure manipulation associated with a cognitive task should impact on affect state and self-esteem of older adults.

Our first goal was to verify whether and how success-failure experimental manipulations associated with a cognitive task differently impact affect states and self-esteem. In order to align the measures of the present investigation to those used by Rosi et al. (2016), we decided to maintain the Positive and Negative Affect Schedule (PANAS; Watson et al., 1988) to assess changes in positive and negative affect states, and the Rosenberg Self-Esteem Scale (RSES; Rosenberg, 1979) as a measure of changes in trait self-esteem. In addition to these questionnaires, we added two new measures: the Affect Grid scale (AG; Russel et al., 1989) and the State Self-Esteem Scale (SSES; Heatherton and Polivy, 1991). The AG scale was used to detect possible changes in other aspects of core affect not tapped by the PANAS, i.e., pleasure and arousal levels (Russel et al., 1989). Pleasure refers to the valence dimension of affective experiences running from pleasing to unpleasing feelings, while arousal assesses the intensity levels of affective reactions from high- to low-arousal levels. The SSES was included to assess changes in state self-esteem for two reasons. First, because previous studies (e.g., Crocker and Luhtanen, 2003; Liu et al., 2014) suggested that state self-esteem is subject to greater fluctuation and it is more affected by the experimental manipulation than trait self-esteem (Crocker et al., 1993). Second, given the lack of age differences in the measure of trait self-esteem in Rosi et al. (2016), we wanted to assess whether age differences occurred in state self-esteem. Moreover, SSES included two subscales that tapped social and performance aspects of state self-esteem. Performance refers to one’s sense of general competence, self-confidence, and efficacy in intellectual abilities and performance. Social state self-esteem refers to how people believe others perceive them. Both failing and having success in our manipulated task may affect both subscales. All the measures were administered before and after the success-failure manipulation. We expected that experiencing success decreases levels of negative affect and increases positive affect, arousal, pleasure and state self-esteem, while failures increase negative affect and decrease positive affect, arousal, pleasure, and state self-esteem. No changes should occur in trait self-esteem.

Our second goal was to examine whether and, if so, how success-failure experimental manipulations differently impact affect states and self-esteem between young and older adults. It is important to note that the success-failure manipulation adopted in the present study appears to provide a feasible indirect test for the reduced physiological reactivity perspective (Levenson et al., 1991). Hence, in line with this perspective and with our previous study (Rosi et al., 2016), we expected that older adults should still display less pronounced emotional responses than young adults in both positive and negative affect states (PANAS) as well as in the levels of arousal (AG). Regarding self-esteem, based on the theoretical consideration outlined above (Crocker and Wolfe, 2001), we expected that state self-esteem in older adults should be less susceptible to manipulations than young adults, in particular following the experience of the failure, while no age differences should occur in trait self-esteem.

## Materials and Methods

### Participants

The study included 100 young adults (age range: 19–30 years; *M*_*age*_ = 24.28, *SD* = 3.06), and 102 older adults (age range: 65–81 years; *M*_*age*_ = 71.61, *SD* = 5.01). The above sample size provides a power greater than 0.8 to detect significant main effects/interaction in the subsequent analyses assuming a medium size (*f* = 0.25) of the effect of the relevant independent variables on the dependent variables considered. Young adults were undergraduate students who received course credit for participating in the study. Older adults were recruited through the local branch of the University of Third Age, located in northern Italy. Participants in each age group were randomly allocated to either the failure or the success condition (see Table 1).

**TABLE 1:** Means values and (standard deviations) of participants’ demographic characteristics.

	**Young adults**		**Older adults**	
	**Success**	**Failure**	**Success**	**Failure**
	**(*n* = 50)**	**(*n* = 50)**	**(*n* = 50)**	**(*n* = 52)**
**Demographic characteristics**				
Age	24.82 (2.98)	23.74 (3.07)	71.86 (5.14)	71.37 (4.92)
Years of education	15.42 (2.34)	14.96 (2.57)	10.74 (4.48)	11.60 (4.56)
Female/male	18/32	23/27	29/21	30/22
MMSE	–	–	27.69 (1.57)	28.29 (1.71)
Vocabulary	39.98 (6.19)	38.92 (5.64)	42.96 (6.07)	44.02 (5.21)

By using the Mini Mental State Examination (MMSE; Folstein et al., 1975), it was ascertained that none of older adults exhibited signs of dementia (i.e., MMSE > 26). A vocabulary test (extracted from the Primary Mental Abilities test – PMA; Thurstone and Thurstone, 1963) was also included in the study as a control variable of crystallized intelligence. All participants completed an informed consent form.

Young adults had significantly more years of education, *F*(1,200) = 61.06, *p* < 0.001, *${\text{η}}_{p}^{2}$* = 0.23, and lower vocabulary scores as compared to the older adults, *F*(1,200) = 24.78, *p* < 0.001, *${\text{η}}_{p}^{2}$* = 0.11. There were no significant differences in either year of educations or vocabulary scores between participants in the success and failure conditions, *F*s ≤ 0.15, *p*s ≥ 0.700, nor any significant interaction with the age factor, *F*s ≤ 1.69, *p*s ≥ 0.195. Demographic characteristics are shown in Table 1.^1^

### Materials^2^

#### Assessment of Affect

In order to assess changes in affect states before and after failure/success induction, participants were administered the Positive and Negative Affect Schedule (PANAS; Watson et al., 1988; Italian version Terracciano et al., 2003) and the Affect Grid (AG; Russel et al., 1989).

The PANAS measures positive and negative affective states. It is a self-report questionnaire consisting of 10 items (affect states) for the Positive Affect scale (PA) and 10 items for the Negative Affect scale (NA). For each affect state, participants rated, on a 5-point Likert scale (anchored at 1 = *very slightly or not all*; 5 = *extremely*), the extent to which they experienced each affect state “at the present moment.” High scores indicate high levels of either positive or negative affect states.

The AG scale is a single-item measure and was used for assessing two dimensions of affective states: Arousal and pleasure. Participants were presented with a 9 × 9 grid, showing the two affective dimensions simultaneously: Horizontally, the scale represents the current pleasantness level (ranging from 1 “*unpleasant feelings*” on the left to 9 “*pleasant feelings*” on the right), while vertically, the scale represents the current arousal level (ranging from 1 “*low arousal*” at the bottom to 9 “*high arousal*” at the top). Participants were required to mark an X in the one square of the grid indicating how they were feeling at that moment. Responses generate two separate scores ranging from 1 (low) to 9 (high) corresponding to the levels of arousal and pleasure.

#### Assessment of Self-Esteem

To assess changes in trait self-esteem and state self-esteem before and after failure/success induction, participants were administered the Rosenberg trait Self-Esteem Scale (RSES; Rosenberg, 1979; Italian version Prezza et al., 1997) and the State Self-Esteem Scale (SSES; Heatherton and Polivy, 1991; Italian version, Bobbio, 2009).

The RSES was used to assess trait self-esteem. It is a self-report questionnaire consisting of 10-item describing a series of statements measuring trait self-worth. Participants have to respond to each item using a 4-point Likert-scale anchored at 1 (*strongly disagree*) and 4 (*strongly agree)*. High scores indicate high levels of trait self-esteem.

State self-esteem^3^ was measured with the SSES, considering the Performance self-esteem and Social self-esteem subscales. Performance self-esteem (composed by 7-items; e.g., “I feel confident about my abilities”) refers to one’s sense of general competence. Social self-esteem (composed by 7-items; e.g., “I am worried about whether I am regarded as a success or failure”) refers to how people believe others perceive them. For each item, participants are asked to rate on a 6-point Likert scale (anchored at 1 = *strongly disagree*; 6 = *strongly agree*) how much they agreed or disagreed with the content of each sentence, thinking about how they feel “at this moment/right now.” High scores indicate high levels of state self-esteem.

### Procedure

Participants were individually tested in a 1-h session. Firstly, participants completed the consent, a demographic questionnaire, the AG, SSES, PANAS and the RSES. Then, participants in the success and failure conditions were presented with a selection of Raven’s Advanced Progressive Matrices. They were informed that this part of the study concerned with assessing their flexibility and mental agility. They were told that they had to perform a test developed to predict their ability to solve problems and extricate themselves from difficult situations. Participants were informed that the average person solves at least 50% of the problems. Participants first practiced with four items, two easy and two difficult ones, and were then asked to estimate, if given ten items of the kind just experienced, how many of these they would expect to solve. Participants’ estimation of their performance before the task showed no significant differences between the success (*M* = 5.03; *SD* = 1.51) and failure (*M* = 4.93; *SD* = 1.70) conditions, *t*(200) = 0.44, *p* = 0.663. The matrices task contained a total of 10 items with a time limit of 50 s to solve each item. Participants in the success condition received 8 easy and 2 difficult matrices, while in the failure condition they received 8 difficult and 2 easy items. Perception of facility/difficulty of items was previously determined in a pilot study. In order to make their experience of success or failure more salient, independently of their actual performance, at the end of the task, a verbal feedback was given to participants indicating that their performance was worse than average in the failure condition and better than average in the success condition. In particular, participants in the success condition were informed that they answered 8 out of 10 items correctly and their performance was above average on the task; participants in the failure condition were told that they were wrong 8 out 10 items and their performance was below average on the task. We examined whether participants had actually experienced a success or failure: subjects in the success condition solved more matrices correctly (*M* = 6.82; *SD* = 2.29) compared to those in the failure condition (*M* = 2.97; *SD* = 1.28), *t*(200) = 14.79, *p* < 0.001.

Immediately following the failure-success manipulated task, participants were asked to complete for a second time the AG, SSES, PANAS and the RSES. Finally, for screening purposes, participants carried out the vocabulary test and, only participants in the older group, the MMSE.

## Results

Initially, in order to assess whether our manipulations were effective in changing affect and self-esteem measure in the expected direction, we compared the means displayed in Table 2 to determine if there were significant differences between post- and pre- manipulation scores. Then, for both classes of measures (affects and self-esteem), we conducted frequentist factorial analyses of variance (ANOVA) with age (young vs. old adults) and manipulation conditions (success vs. failure) as between-groups independent variables. In these analyses, to allow a direct comparison in the magnitude of the changes in affect and self-esteem measures between the success and failure conditions, we considered absolute changes in post- minus pre-manipulation scores. Average absolute change scores are displayed in Table 3. In the ANOVA analyses, we were interested in the main effect of age in order to verify whether older adults show reduced affective and self-esteem responses to the failure/success manipulation. We also considered the main effect of condition in order to verify whether the manipulation produced stronger responses on affect and self-esteem as a function of failures or success experiences. Finally, we were interested in the two-way interaction Age by Condition, in order to verify whether a differential effect of the success/failure manipulation occurs between young and older adults.

**TABLE 2:** Summary of Bayesian analysis for the effect of manipulation on affect states and self-esteem variables separately for success and failure conditions.

	**Successes**				**Failures**			
	**Mean diff post-pre**	**95% CI**	**Cohen’s *d***	**BF**	**Mean diff post-pre**	**95% CI**	**Cohen’s *d***	**BF**
**Affect state**								
PANAS – positive affect	0.161	0.094, 0.228	0.476	>100	–0.223	−0.296, −0.149	0.591	>100
PANAS – negative affect	–0.112	−0.158, −0.066	0.478	>100	0.098	0.027, 0.169	0.272	3.83
AG – arousal	0.107	0.060, 0.153	0.455	>100	–0.016	−0.065, 0.032	0.066	0.14
AG – pleasure	0.094	0.057, 0.132	0.496	>100	–0.169	−0.221, −0.117	0.641	>100
**Self-esteem**								
RSES	0.044	0.002, 0.086	0.209	0.88	–0.014	−0.058, 0.031	0.06	0.13
SSES	0.225	0.154, 0.296	0.631	>100	–0.28	−0.40, −0.16	0.46	>100

*Means diff post-pre, absolute means differences between post- and pre- manipulation scores; BF, Bayesian Factor; PANAS, positive and negative affect schedule; AG, affect grid; RSES, Rosenberg self-esteem scale; SSES = state self-esteem scale.*

**TABLE 3:** Pre- and post-experimental manipulation scores, and absolute means differences between post- minus pre- manipulation scores (see text for an extended explanation of absolute scores’ computation) as a function of condition (success vs. failure) and age groups (young vs. old).

	**Young adults**						**Older adults**					
	**Success**			**Failure**			**Success**			**Failure**		
	**Pre**	**Post**	**Post-pre**	**Pre**	**Post**	**Post-pre**	**Pre**	**Post**	**Post-pre**	**Pre**	**Post**	**Post-pre**
**Affect state**												
PANAS – positive affect	3.37 (0.59)	3.59 (0.62)	0.22 (0.37)	3.20 (0.60)	2.95 (0.64)	0.25 (0.38)	2.94 (0.48)	3.03 (0.50)	0.09 (0.29)	3.01 (0.49)	2.82 (0.50)	0.20 (0.37)
PANAS – negative affect	1.39 (0.44)	1.22 (0.40)	0.17 (0.21)	1.50 (0.60)	1.65 (0.74)	0.15 (0.37)	1.25 (0.27)	1.20 (0.27)	0.06 (0.25)	1.26 (0.31)	1.29 (0.38)	0.03 (0.36)
AG – arousal	0.60 (0.24)	0.75 (0.20)	0.15 (0.23)	0.56 (0.23)	0.53 (0.22)	0.03 (0.24)	0.56 (0.21)	0.62 (0.21)	0.06 (0.24)	0.56 (0.25)	0.56 (0.21)	0.00 (0.25)
AG – pleasure	0.73 (0.20)	0.84 (0.14)	0.11 (0.15)	0.69 (0.23)	0.52 (0.24)	0.17 (0.28)	0.71 (0.22)	0.79 (0.15)	0.08 (0.23)	0.77 (0.20)	0.60 (0.26)	0.17 (0.26)
**Self-esteem**												
RSES	3.20 (0.39)	3.26 (0.39)	0.06 (0.21)	3.03 (0.43)	3.03 (0.41)	0.00 (0.20)	3.11 (0.34)	3.13 (0.37)	0.02 (0.21)	3.26 (0.35)	3.23 (0.36)	0.03 (0.26)
SSES	4.61 (0.60)	4.88 (0.59)	0.27 (0.29)	4.45 (0.72)	4.16 (0.78)	0.29 (0.53)	4.53 (0.53)	4.71 (0.52)	0.18 (0.41)	4.74 (0.60)	4.46 (0.78)	0.28 (0.61)

*Scores in parenthesis refers to Standard Deviation; PANAS, positive and negative affect schedule; AG, affect grid; RSES, rosenberg self.*

Furthermore, for each of the above analyses, we also calculated Bayes factors (BF) using JASP’s (JASP Team, 2018) default setting for the *a priori* distribution of the parameters of interest (Wagenmakers et al., 2018). BF provides a statistical index quantifying the degree of evidence in favor of the alternative versus the null hypothesis or vice-versa. Values greater than 1 indicate propensity to consider the alternative hypothesis more likely than the null. Conversely values smaller than 1 indicate the opposite. BF comprised between 1/3 and 3 are inconclusive/anecdotal; values comprised between 3 and 10 provides moderate evidence for the alternative hypothesis (conversely those between 1/3 and 1/10, for the null hypothesis). Larger (smaller) values indicate stronger levels of evidence (e.g., Lee and Wagenmakers, 2013).

A preliminary inspection of the data (see Table 2 for the statistical values), investigating the effect of manipulation on affect states and self-esteem variables separately for success and failure conditions, showed that both success and failure manipulations induced robust changes in the expected directions for the dependent variable used (e.g., positive affect increased following a success experience and decreased following a failure experience). The only exception was the measures of trait self-esteem (RSES) showing that there was not an influence of either the success or the failure manipulation. The failure manipulation also had limited or no impact on the levels of arousal (AG scale).

Pre- and post-experimental manipulation scores, and post- minus pre-manipulation scores mean ratings on affect states and self-esteem measures are reported in Table 3 as a function of age groups and conditions.

### Positive Affect (PANAS)

Results from the positive affect scale of PANAS showed a main effect of age approaching significance, *F*(1,198) = 3.51, *p* = 0.062, ${\text{η}}_{p}^{2}$ = 0.02 (BF = 0.76) with young adults having higher scores than older adults. The Bayesian analysis, on the other hand, is more supportive of a lack of age effect. Notice that also assuming a half-normal distribution of the effect of age with a mean of zero and a standard deviation of 0.37 (as *a priori* distribution of the age effect parameter based on Rosi et al., 2016 data), the BF would be 1.42, that while in the direction of an age effect would still be inconclusive. Neither the Condition effect, *F*(1,198) = 1.54*, p* = 0.217, ${\text{η}}_{p}^{2}$ = 0.01, (BF = 0.31), nor the Condition by Age interaction were significant, *F*(1,198) = 0.64, *p* = 0.425, ${\text{η}}_{p}^{2}$ = 0.02 (BF = 0.27). This interaction outcome, and particularly the Bayesian analysis, provides support for no differential effect of the success/failure manipulation across ages.

### Negative Affect (PANAS)

Results from the negative affect scale of PANAS showed a significant main effect of age, *F*(1,198) = 7.38, *p* = *0.007*, ${\text{η}}_{p}^{2}$ = 0.036 (BF = 4.86) with young adults having higher scores than older adults. Notice that also assuming a half-normal distribution of the effect of age with a mean of zero and a standard deviation of 0.12 (as *a priori* distribution of the age effect parameter based on Rosi et al., 2016 data), the BF would be 25.9, that supports an age effect. The Condition effect was not significant, *F*(1,198) = 0.09 *p* = 0.762, ${\text{η}}_{p}^{2}$ = 0.001 (BF = 0.16) nor the Condition by Age interaction, *F*(1,198) = 0.004, *p* = 0.947, ${\text{η}}_{p}^{2}$ = 0.001 (BF = 0.21). Overall, these results suggested that both manipulations had the same impact on negative affect, and that there was no differential effect of the success/failure manipulation across ages.

### Arousal (Affect Grid Scale)

Analysis from the arousal scale of the AG revealed only a significant main effect of condition, *F*(1,198) = 7.15, *p* = 0.008, *${\text{η}}_{p}^{2}$* = 0.035 (BF = 4.17), indicating that the success condition had stronger responses on levels of arousal than the failure condition. Neither the main effect of age, *F*(1,198) = 3.07, *p* = 0.081, ${\text{η}}_{p}^{2}$ = 0.01 (BF = 0.63), nor the Condition by Age interaction were significant, *F*(1,198) = 0.79, *p* = 0.375, ${\text{η}}_{p}^{2}$ = 0.004 (BF = 0.30). Anecdotal evidence is provided to the lack of age differences and moderate evidence to the lack of a differential effect of the experimental manipulation on different age groups.

### Pleasure (Affect Grid Scale)

Analysis from the pleasure scale of the AG revealed only a significant main effect of condition, *F*(1,198) = 5.24, *p* = 0.023, *${\text{η}}_{p}^{2}$* = 0.03 (BF = 1.78). Neither the main effect of age, *F*(1,198) = 0.33, *p* = 0.564, *${\text{η}}_{p}^{2}$* = 0.002 (BF = 0.18), nor the Condition by Age interaction were significant, *F*(1,198) = 0.10, *p* = 0.76, *${\text{η}}_{p}^{2}$* = 0.001 (BF = 0.24). Overall, while the output of the standard ANOVA about the main effect of condition seems to imply that stronger responses to failures occurred, the Bayesian analysis only lends anecdotal support to this conclusion, while moderate support is provided to the lack of age differences and of a differential effect of the experimental manipulation on different age groups.

### Trait Self-Esteem (Rosenberg Self-Esteem Scale)

Results from the RSES revealed neither a significant main effects of age, *F*(1,198) = 0.08, *p* = 0.783, *${\text{η}}_{p}^{2}$* = 0.001 (BF = 0.16); nor of condition, *F*(1,198) = 0.98, *p* = 0.324, *${\text{η}}_{p}^{2}$* = 0.005 (BF = 0.24), nor of the Condition by Age interaction, *F*(1,198) = 1.32, *p* = 0.252, *${\text{η}}_{p}^{2}$* = 0.007 (BF = 0.39). Overall, there is moderate evidence for the lack of both conditions, age differences, and of the differential effect of the experimental manipulation on different age groups.

### State Self-Esteem (State-Self Esteem Scale)

Concerning the analysis of the SSES, results showed neither a significant main effect of age, *F*(1,198) = 0.38, *p* = 0.539, *${\text{η}}_{p}^{2}$* = 0.002 (BF = 0.18), nor of condition, *F*(1,198) = 0.61, *p* = 0.436, *${\text{η}}_{p}^{2}$* = 0.003 (BF = 0.2). The interaction was also not significant, *F*(1,198) = 0.34, *p* = 0.564, *${\text{η}}_{p}^{2}$* = 0.002 (BF = 0.008). Overall, it appears that the null hypothesis is the most plausible option for both main effects and the interaction.

## Discussion

The first aim of the present study was to investigate the extent to which experimentally induced experiences of success and failure could differentially change affect states and levels of self-esteem. Particularly, we attempted to generalize the outcome of the Rosi et al. (2016) study to a situation where failure and success were experienced while performing a demanding cognitive task. Overall we found that the success-failure manipulation adopted was effective in inducing robust changes on all affect states measures and on state self-esteem in the expected directions. In particular, regarding changes in affect states, we found that the success condition induced a decrement in negative affect scores and an increment in positive affect, levels of pleasure and arousal, while the failure condition induced only a decrement in positive affect scores and in levels of pleasure. These findings are in line with previous studies (e.g., Krohne et al., 2002; Nummenmaa and Niemi, 2004; Franken et al., 2006; Rosi et al., 2016) showing that success-failure manipulation is effective in determining changes not only in positive and negative affect states but also in the other two dimensions of affect states, i.e., pleasure and arousal.

Regarding self-esteem,^4^ different results were obtained for trait and state self-esteem. Indeed, our results showed, as expected, that our manipulation did not affect the measure of trait self-esteem (RSES), while the impact was robust on the measure of state self-esteem (SSES). In particular, the success condition induced an increment of state self-esteem, while the failure condition induced a decrement. This pattern of results suggests that state self-esteem is subject to greater fluctuation than trait self-esteem and that it is actually more sensitive to failure and success experiences (Heatherton and Wyland, 2003).

Interestingly, looking at the different impact of the failure and success conditions on affect states changes, we found that the success condition primarily influenced the levels of arousal, while the failure condition had influence on levels of pleasure. Our findings suggest that the intensity of affective reactions is more influenced by experiencing a success, while the valence of emotion states is more affected by experiencing failures.

The effect of failures and successes on affect state and self-esteem is not surprising; in everyday life affect state and self-esteem are indeed linked: People in positive affect tend to feel good about themselves and people who lack self-worth are generally in negative mood (e.g., Lyubomirsky et al., 2006). Not surprisingly, some studies used self-esteem as an index of global positive emotion or psychological well-being (e.g., Ryff, 1989).

With respect of the second aim, we were interested to investigate whether success-failure experimental manipulations differently impact on affect states and self-esteem between young and older adults. Regarding changes in positive affect, contrary to Rosi et al. (2016) study in which there was evidence of an age effect, in the present study the success-failure manipulation did not differentially affect young and older adults. Conversely, we detected a strong main effect of age with young being more severely affected than older adults in changes in negative affect following both successes and failures. This result is in line with previous studies where it was found that levels of negative affect tend to decrease in aging (e.g., Charles et al., 2001). However, contrary to our expectations, we did not detect a significant interaction between age and condition. Neither differential age effects occurred on arousal and pleasure levels, nor there was evidence of a main effect of age. As indicated in the introduction, it is still under debate whether older adults tend to regulate their affect states in order to avoid negative affect responses and to optimize the positive ones (Gross et al., 1997), as suggested by the SST (Carstensen et al., 1999) and the affect-regulatory perspectives (Gross et al., 1997; Labouvie-Vief and Medler, 2002; Blanchard-Fields et al., 2004), or whether they respond to emotional stimuli with lower emotional impact than younger adults due to an age-related decline in physiological reactivity for emotional stimuli (Levenson et al., 1991). Results of the present study do not appear to support the SST account nor affect-regulatory perspectives given that for all the affect measures used there was moderate evidence of a comparable impact of successes and failures in both age groups. There is anecdotal to moderate evidence of reduced responses to both success and failure manipulations in the PANAS that would lend some support to the age-related decline in physiological reactivity view. According to this account, older adults should show less pronounced affective changes to both positive (success) and negative (failure) experiences as compared to younger adults (see also Röcke et al., 2009). This consideration is quite speculative since in the present study we did not collect physiological data on reactivity to emotional situations. Moreover, it is worth noticing that we used the Affect Grid in order to measure the arousal and pleasure levels associated with the experience of positive and negative affect. While we found that success increased levels of arousal and pleasure, and that failure only reduced levels of pleasure, we did not detect age differences. The moderate evidence about the absence of age differences on pleasure and, anecdotal on arousal, may, thus provide some evidence against the physiological reactivity view. While this may be the case, we would like to point out that the above outcome could be accounted for by the characteristics of the Affect Grid scale. Indeed, AG is a single-item measure of core affect that examines the simultaneous experience of different dimensions of affect states. It is worth considering that there is no clear indication of the extent to which the arousal dimension of AG is associated to physiological activity. Indeed, the arousal dimension of AG is rather an index of reported subjective feeling (Russel et al., 1989). There is, therefore, a pressing need for future research, focusing on measuring physiological reactivity during success-failure manipulations, to clarify whether older adults’ physiological reactivity is reduced as compared to younger adults.

Regarding age differences in levels of self-esteem, there was no support for a differential effect of the success/failure manipulation across age groups.

These pattern of results suggested that failing or having a success in a demanding cognitive task achieved the same effect on affect states and on state self-esteem both in young and older adults. We could hypothesize that the absence of age differences was due to the kind of manipulation adopted. Indeed, not only older adults are interested in succeeding or not failing in a cognitive task, given their negative beliefs about their cognitive abilities (Chasteen et al., 2005), but also for young adults, who are still close to academic experiences, having a success or a failure in a task similar to academic performance has a strong impact on their affect states and self-worth (Crocker and Wolfe, 2001; Meier et al., 2011). However, we are not sure that the two age groups have the same motivation to success or to failure in the task. There is evidence that older adults tend to have greater stability in self-esteem (e.g., Meier et al., 2011). A greater balance in self-esteem in the older adults group may have flattened their emotional reactions to success/failure compared to the young adult group. Future studies should assess the level of motivation of participants and testing whether adopting a different success-failure manipulation task, such as a social perception task (e.g., McFarland and Buehler, 1997; Nummenmaa and Niemi, 2004), should be more relevant and be more motivating for older adults than younger adults. This could help to further our understanding on the nature of age differences in self-esteem and affective states associated with success and failure events.

Overall, we generalized the results of Rosi et al. (2016) and provided further support with respect to the following issues: (a) The success-failure manipulation induced changes on positive/negative affect and also on levels of arousal/pleasure; (b) the success-failure manipulation affected state self-esteem but not trait self-esteem. On the other hand, we found that older and young adults were affected to the same extent by experiences of successes and failures.

## Conclusion

In conclusion, our study provided a laboratory-based assessment of the impact of success and failure on affect and self-esteem in young and older adults. The key finding of the present study is that overall older and young adults are comparably affected by failure and success. It is important to keep in mind that the emotional charge of events experienced in the laboratory may well be more subdue than those associated to real life events, thus we may well expect that laboratory manipulations may be less impactful than real-life emotional experiences (Oerlemans et al., 2011), where the need to regulate emotions to deal with positive/negative events may be more substantial. An important task for future research is to examine not only age differences in emotional charge associated to real life events, but also to investigate how the wide range of life experiences in older adults could impact on affect and self-esteem differently compared younger adults. Finally, a new study should involve a larger number of participants in order to generalize and replicate these results.

From a theoretical perspective, the success-failure manipulation adopted was effective in inducing changes in affect states and state self-esteem. Regarding the effect of age, our results did not support that older adults are differently influenced by the manipulations compared to younger adults. To refine our understanding on this issue, future studies may attempt to use different experimental procedures to induce successes and failures in conjunction with a larger set of measures of affect and self-esteem.

## Ethics Statement

This study was carried out in accordance with the recommendations of the Italian Association of Psychology with written informed consent from all subjects. All subjects gave written informed consent in accordance with the Declaration of Helsinki. The protocol was approved by the Ethical Committee of the University of Pavia/IUSS.

## Author Contributions

AR collected and coded the data, helped in the designing of the study, performed the analyses, and had a major role in the writing of the manuscript. EC designed the study and had a major role in the writing of the manuscript and revising it critically for the important intellectual content. NG and TV collaborated to evaluate and edit the submitted manuscript. FVV contributed to the statistical analyses and the writing of the revision. RR designed the study, responsible for the statistical analyses, and had a major role in the writing of the manuscript and revising it critically for the important intellectual content.

## Conflict of Interest Statement

The authors declare that the research was conducted in the absence of any commercial or financial relationships that could be construed as a potential conflict of interest.

## Footnotes

1 Younger adults were significantly more male, while older adults were significantly more female, χ^2^(1) = 5.73, *p* = 0.017. Given these gender differences across the age groups, all statistical analyses were also controlled for gender and the results did not change.

2 In order to ensure equivalence of questionnaires’ factor structure across age groups, we provided in the Supplementary Material the results of the analyses conducted on the two samples of participants for the PANAS, AG, RSES, and SESS.

3 For the SESS, the loading factors of Social and Performance subscales on young and older adults were not equivalent. Hence, in order to ensure equivalence of questionnaire’s factor structure across age groups, we have conducted the analyses by combining Social and Performance subscales in a single scale of state self-esteem (see Supplementary Table S4).

4 Previous studies found that men appear to be less self-esteem contingent than women (e.g., Sargent et al., 2006). We found gender differences across young and older adults representing a limitation of the present study. However, we have conducted all analyses controlling for gender and the results did not change.
