# Supplementary material for: The Impact of Failures and Successes on Affect and Self-Esteem in Young and Older Adults
Source: Front Psychol. 2019 Aug 6;10:1795. doi: 10.3389/fpsyg.2019.01795 (PMC6691139; doi:10.3389/fpsyg.2019.01795)
Supplement: Supplementary file 1 [file Data_Sheet_1.PDF]

The impact of failures and successes on affect and self-esteem in young and older adults

Online supplementary material

Table S1

Results of the Confirmatory Factor Analyses conducted on the two samples of participants for the Positive and Negative Affect Scale (PANAS).

Items	Factors			
	Young adults		Older adults	
	Factor 1	Factor 2	Factor 1	Factor 2
Distressed (NA)	.809		.430	
Upset (NA)	.798		.659	
Nervous (NA)	.792		.655	
Afraid (NA)	.772		.665	
Scared (NA)	.762		.561	
Jittery (NA)	.687		.687	
Irritable (NA)	.594		.519	
Guilty (NA)	.531		.437	
Hostile (NA)	.528		.396	
Ashamed (NA)	.516		.218	
Strong (PA)		.736		.641
Inspired (PA)		.725		.600
Determined (PA)		.718		.725
Enthusiastic (PA)		.718		.662
Active (PA)		.711		.664
Interested (PA)		.671		.673
Excited (PA)		.610		.436
Attentive (PA)		.514		.663
Proud (PA)		.510		.490
Alert (PA)		.487		.599
Cronbach Alpha	.87	.84	.71	.82
Inter-item correlation	.83	.74	.51	.68

Note. NA = Negative Affect Scale; PA = Positive Affect Scale

Table S2

Results of Correlation analysis conducted on the two samples of participants between the Arousal and Pleasure dimensions of the Affect Grid Scale (AG)).

	**Arousal Pre**		**Arousal Post**		**Pleasure Pre**		**Pleasure Post**	
	Young	Old	Young	Old	Young	Old	Young	Old
Arousal Pre	-	-	.44**	.37***	-.41***	-.22**	-.18	-.16
Arousal Post	.44***	.37***	-	-	-.02	-.08	.25*	-.25*
Pleasure Pre	-.41***	-.22*	-.02	-.08	-	-	.38***	.27**
Pleasure Post	-.18	-.16	.25*	-.25*	.38***		-	-

Note. ***p < .001; ** p < .01: *p < .05

Internal consistency reliability and factorial analysis cannot be computed for a single-item scale. In order to be sure that the pattern of correlation was similar between young and older adult groups, we run correlations analyses between the two sub-dimensions of arousal and pleasure. The trend of correlation between arousal pre-manipulation and pleasure pre-manipulation was similar in young and older adults, as well as the correlation between pre-manipulation and post-manipulation scores.

Table S3

Results of the Confirmatory Factor Analyses conducted on the two samples of participants for the Rosenberg Self Esteem Scale (RSES).

**Items**	**Factors**	
	Young adults	Older adults
	Factor 1	Factor 1
I certainly feel useless at times.	.773	.702
I take a positive attitude toward myself.	.748	.486
I feel I do not have much to be proud of.	.719	.600
At times I think I am no good at all.	.712	.615
On the whole, I am satisfied with myself.	.693	.504
All in all, I am inclined to feel that I am a failure.	.635	.630
I am able to do things as well as most other people.	.509	.657
I feel that I am a person of worth, at least on an equal plane with others.	.435	.690
I feel that I have a number of good qualities.	.418	.376
I whish I could have more respect for myself.	.337	.422
Cronbach Alpha	.79	.76
Inter-item correlation	.65	.57

Table S4

Results of the Confirmatory Factor Analyses conducted on the two samples of participants for the State Self-Esteem Scale (SSES).

**Items**	**Factors**	
	Young adults	Older adults
	Factor 1	Factor 1
I feel self-conscious.	.723	.668
I am worried about what other people think of me.	.707	.542
I feel like I am not doing well.	.699	.299
I feel concerned about the impression I am making.	.682	.722
I feel inferior to others at this moment.	.673	.635
I am worried about looking foolish.	.661	.542
I feel that I have less scholastic ability right now than others.	.643	.566
I feel confident about my abilities.	.624	.400
I am worried about whether I am regarded as a success or failure.	.595	.438
I feel frustrated or rattled about my performance.	.513	.416
I feel displeased with myself.	.406	.477
I feel confident that I understand things.	.344	.317
I feel as smart as others.	.335	.139
I feel that I am having trouble understanding things that I read.	.281	.565
Cronbach Alpha	.83	.73
Inter-item correlation	.59	.43
